# Blind spots of psychotherapists? Implicit and explicit mental illness stigma in psychotherapists, psychology students, and laypersons

**DOI:** 10.3389/fpsyg.2026.1759801

**Published:** 2026-02-27

**Authors:** Elena Stoll, Greta Jakobsen, Susanne Hörz-Sagstetter, Emily Nething, Samuel Tomczyk

**Affiliations:** 1 Department of Health and Prevention, Institute of Psychology, University of Greifswald, Greifswald, Germany; 2 Department of Clinical Psychology and Psychotherapy, Psychologische Hochschule Berlin, Berlin, Germany; 3 Department of Psychosocial Medicine, Institute of Medicine Psychology and Medicine Sociology, University Medicine Rostock, Rostock, Germany

**Keywords:** help seeking, implicit association task, mental health professionals, mental health stigma, mental illness stigma, clinical psychologists, psychotherapists

## Abstract

**Background:**

While explicit mental illness stigma in mental health professionals is often lower compared to laypersons, implicit mental illness stigma is not. However, implicit mental illness stigma may negatively affect treatment processes and outcomes, for example, via over-diagnosis, yet few studies examine such attitudes held by psychotherapists/clinical psychologists. We explored whether psychotherapists differ from psychology students and laypersons in their explicit or implicit mental illness stigma.

**Methods:**

We created a Single Category Implicit Association Task to measure implicit stigma. We tested it in a sample of 108 academic laypersons without a psychological background (students and graduates) and in 82 psychology students and psychotherapists. We also measured explicit mental illness stigma and, for example, social desirability via established self-report surveys.

**Results:**

Psychotherapists showed significantly lower explicit mental illness stigma than graduate academics with a medium sized effect. The groups did not differ in their implicit, negatively biased, mental illness stigma. Social desirability was connected to implicit, but not explicit attitudes.

**Discussion:**

The findings are in line with the Dual Attitude Model: explicit mental illness stigma in the psychotherapy subsample may reflect especially positive attitudes towards mental illness due to, for example, specific aspects of psychotherapeutic training or professional values, while implicit mental illness stigma may reflect negative attitudes deeply embedded in society that are resistant to change.

## 1 Introduction

Although more than one in four people can be affected by a mental illness (Jacobi et al., 2014, 2015), only 18.9% of those affected seek professional help (Mack et al., 2014). One of the barriers for seeking help is mental illness stigma (Clement et al., 2015; Gallimore et al., 2023; Schomerus et al., 2019). There are complex interactions between mental illness stigma and help seeking and there is some evidence that positive psychotherapeutic treatment experience is connected to lower mental illness stigma (McLaren et al., 2023). But people living with mental illness (PLMI) continuously experience the burden of stigmatization of their illness (e.g., Hazell et al., 2022; Ren et al., 2025), even in the healthcare system which is supposed to help them overcome their mental illness struggles (Lauber et al., 2006; O’Connor and Yanos, 2023; Thornicroft et al., 2007; Ungar et al., 2016). There is some evidence that psychiatrists show more explicit mental illness stigma than psychologists (Del Olmo-Romero et al., 2019; Lauber et al., 2006). Besides, there are findings that clinical psychologists tend to stigmatize severe mental illness, like psychotic disorders or personality disorders (Gonzales et al., 2021; O’Connor and Yanos, 2023). Research on mental illness stigma among mental health professionals (MHP) is scarce, but research examining mental illness stigma specifically among (psychologically trained) psychotherapists/clinical psychologists is even more limited (López-Aybar et al., 2024; López-Aybar and Gonzales, 2024; Schomerus and Lüders, 2023).

First, it is important to discern implicit and explicit aspects of stigma. Link and Phelan (2001) define stigmatization as a process that involves first, labelling undesirable human differences, which second, leads to stereotypes. These stereotypes operate in a “preconscious, automatic way” (p. 369) to save cognitive resources. In the third step of the stigma process, labels separate “us” from “them,” and in the last step, “the labelled person experiences status loss and discrimination” (p. 370). Following this definition, mental illness stigma can comprise conscious and unconscious processes, which are supposed to be captured differently by explicit and implicit measures, although there is still a lack of theoretical ground to explain the nature and the differences between both measures (Greenwald and Banaji, 2017). Generally, explicit measures of attitudes are assessed via, for example, self-report questionnaires. For example, stigma of mental illness of MHP is examined using existing questionnaires like Opinions about Mental Illness Scale (see, e.g., Kopera et al., 2015). Implicit measures are conventionally assessed via computer tasks like the Implicit Association Task (IAT; Greenwald et al., 1998) or Go-No-Go-Association Task (GNAT; Nosek and Banaji, 2001) that are supposed to measure the strength between the associations of attitudes by recording the response times.

Mental illness stigma of MHP is usually examined using explicit measures like self-report questionnaires. However, some scholars examine both explicit and implicit mental illness measures, using different implicit tasks like IAT or GNAT to assess implicit mental illness stigma (e.g., Kopera et al., 2015; Peris et al., 2008; Sandhu et al., 2019; Stull et al., 2013; Wang et al., 2016). Both implicit tasks, IAT and GNAT, rest on the assumption that the variation in response times to presented categories, and to their positive or negative evaluations, reflects the degree to which individuals implicitly endorse or do not endorse certain attitudes toward those categories. While the IAT operates with two contrasting categories to explore stigma (e.g., black vs. white, female vs. male), which are evaluated in several trials (Greenwald et al., 1998), GNAT has only one target category requiring a response, respectively distractor items, requiring a response inhibition (Nosek and Banaji, 2001). Using the IAT, Peris et al. (2008) found that explicit mental illness stigma was connected to negative assumptions about the treatment outcome, while implicit mental illness stigma was connected to over-diagnosis. Wang et al. (2016) discovered that medical students’ explicit and implicit mental illness stigma (measured with IAT) did not differ compared to a non-medical student sample. But while explicit mental illness stigma was significantly reduced after a psychiatric clerkship in the medical student sample, implicit mental illness stigma values did not change. In contrast to Peris et al. (2008) and Wang et al. (2016), Kopera et al. (2015) created a GNAT (Nosek and Banaji, 2001) as a measure of implicit attitudes, arguing that there is no suitable contrast category for mental illness. They found differences between the explicit stigmatizing attitudes in a sample of professionals (psychiatrists and psychotherapists with at least 2 years’ experience in clinical work) as compared to first-year medical students: explicit mental illness stigma was lower in the professional group. Regarding implicit mental illness stigma, they found no significant differences between the groups. Wang et al. (2016) excluded participants with personal experience of mental illness, Kopera et al. (2015) excluded laypersons with personal relationships with PLMI. These exclusion criteria are understandable since contact to PLMI and personal experience with mental illness are factors that can reduce mental illness stigma (Hackler et al., 2016). But it remains unclear whether the difference in mental illness stigma that exists between mental health (e.g., psychology) students and other mental health professionals (e.g., psychotherapists) on the one hand and laypersons without psychological or medical training on the other hand evolves due to more experiences with PLMI or due to other differences.

In Germany, it is more common that psychotherapists have a psychological and not a medical background (Ärzteblatt, D.Ä.G., Redaktion Deutsches, 2011a; Statistisches Bundesamt, 2021). Based on the requirements for training, German psychotherapists can be compared to clinical psychologists in the U.S. Kopera et al. (2015) do not differentiate their sample based on profession: it remains unclear whether the group of psychotherapists consists of psychologists or of medical doctors; however, the latter seems to be the more common case in Poland (Ärzteblatt, D.Ä.G., Redaktion Deutsches, 2011b), where the study was conducted. Nevertheless, the studies by Kopera et al. (2015) as well as other cited works underscore that understanding whether and why (psychologically trained) psychotherapists hold less mental illness stigma could provide improvement in patient care, e.g., in designing anti-stigma interventions for professional groups working with PLMI. Overall, implicit stigma in psychotherapists is an underexplored topic (e.g., O’Connor and Yanos, 2021). It remains unclear whether the dissociation between explicit and implicit attitudes, as reported in studies cited for other MHPs, also applies to psychotherapists and clinical psychologists.

## 2 Methods

### 2.1 Study objectives and hypotheses

In our study, we examined whether the implicit and explicit attitudes towards mental illness between undergraduate psychology students and psychotherapists on the one hand and academic and student laypersons on the other hand differ.

We hypothesized an at least satisfactory level of internal reliability of the SC-IAT (*H1*) and small to medium correlations with the explicit questionnaires on which the SC-IAT was based. These correlations are expected to be higher than the correlation with another stigma questionnaire, on which the VASI was not based (*H2*). We hypothesized no correlation between SC-IAT and a social desirability questionnaire (*H3*).

Moreover, we expected a significant difference between the groups regarding explicit mental illness stigma (*H4*), explicit stigma levels of psychotherapists should be lower than those of psychology students (*H4a*) and the explicit stigmatizing attitudes of a “psych” sample (comprising psychotherapists and psychology students) should be lower than those of the “academic laypersons” sample (comprising graduate academics and non-psychology students) (*H4b*). Regarding implicit mental illness stigma, we expect significant differences between the four subgroups (*H5*): Both subgroups in the “psych” sample are expected not to differ (*H5a*), but we expect both “psych” subgroups to show lower negative implicit attitudes than the layperson subgroups (*H5b*).

### 2.2 Study design and ethics

We developed two online cross-sectional surveys, one for the academic sample: laypeople graduate academics and laypeople students; and one for the “psych” sample: psychotherapists (in training) and psychology students.

For the implementation of the empirical studies in the project, approval was obtained from the local ethics committee. Participants had to give their consent by agreeing to an online data protection form before they could start completing the survey.

Our reporting follows the TREND Guideline.

### 2.3 Participants and procedure

In previous research findings effect sizes regarding explicit and implicit differences in mental illness stigma between laypersons and MHP varied between small and large effects (Kopera et al., 2015; Peris et al., 2008; Sandhu et al., 2019; Stull et al., 2013), thus the sample size calculation was based on a medium effect of ANOVA and medium correlation sizes, in regard to correlations between explicit and implicit measures (Greenwald and Banaji, 2017). Based on these effect size parameters, *α* = 0.05 and 1-*β* = 0.80, G*Power (Faul et al., 2009) calculated a minimum sample size of N = 180 for the total sample. The recruiting process lasted from the middle of December 2024 to the end of April 2025.

Participants for the “psych” sample were recruited via private and university mailing lists. The target groups were undergraduate psychology students and psychotherapists (including psychotherapy trainees with at least 2 years of training). Participants for the layperson sample were recruited via private mailing lists. The target groups were academics, defined as people who have at least begun their studies at a higher education institution. In this way, we controlled for possible effects of higher education on mental illness stigma (e.g., Girma et al., 2013; Kim et al., 2019; Lo et al., 2021) of the “psych” sample, contrasting it to an academic layperson, “non-psych,” sample.

Explicit and implicit measures were both presented via an online questionnaire developed on the platform SoSci Survey (version 3.5.01) (Leiner, 2024). The implicit measure, the SC-IAT, was programmed using a dedicated tool on this platform. Participants could only participate in the survey with a keyboard-enabled device, as this was a prerequisite for the correct completion of the implicit test. The presentation of both explicit and implicit tasks was randomized. The implicit task was pretested with psychology and non-psychology students and psychotherapists and non-psychotherapists. In this process some stimuli items from the SC-IAT were removed, so most items have been derived from VASI (see Table 1) and the instruction was adapted and expanded to enable better understanding of the implicit task. The task duration in pre-tests did not exceed 15 min. Both groups were incentivized with the participation in a raffle of book vouchers in case of fully completed task and questionnaires. Additionally, undergraduate students of psychology were offered credit points: Undergraduate psychology students at German universities are typically required to participate as research subjects as part of their curricula. They can choose from a range of different studies in which to participate. Participation is anonymous, and the results cannot be linked to individual participants. Voluntariness and the absence of negative consequences for non-participation were explicitly emphasized for all participants. There was no blinding; all participants were informed that the study examined explicit and implicit attitudes toward mental illness.

**Table 1:** Final SC-IAT Items and their evaluative dimensions derived from VASI and SSOSH: original German and translated English versions.

Stimuli items	German (original)	English (translation)
Target items	Hilfe von Psychotherapeut*in Leben mit psychisch Kranken psychisch erkranktes Familienmitglied psychisch erkrankte Nachbar*in psychisch erkrankte Kolleg*in eigene psychische Erkrankung psychisch erkrankte im Stadtbild	Help from a psychotherapist living with a mentally ill person mentally ill family member mentally ill neighbour mentally ill colleague personal mental illness mentally ill people in the cityscape
Positive evaluative items	fleißig stark Angenehm Selbstbewusst selbstbestimmtes Leben Statusgewinn bereichernd behaglich harmlos intelligent verantwortungsvoll	diligent strong pleasant self-confident self-determined life status gain enriching comfortable harmless intelligent responsible
Negative evaluative items	faul schwach unangenehm unsicher fremdbestimmtes Leben Statusverlust energie-raubend unbehaglich gefährlich dumm verantwortungslos	lazy weak unpleasant insecure externally determined life loss of status energy-sapping uncomfortable dangerous stupid irresponsible

### 2.4 Outcomes

Choosing an implicit measure, a few methodological aspects should be considered. There are issues pertaining to the GNAT’s reliability (James, 2018; Williams and Kaufmann, 2012) and Kopera et al. (2015) do not report the reliability of their test. Arguing that IAT needs a suitable comparison category, they seem to disregard the adaptation of IAT using a single category, the Single Category Implicit Association Task SC-IAT (Karpinski and Steinman, 2006). For instance, SC-IAT was used to assess negative outcome expectations towards psychotherapy (Seewald et al., 2023) or the self-concept of personality (De Cuyper et al., 2017) and shows satisfying reliability in the cited studies. Moreover, when comparing implicit and explicit attitudes, one would assume a parallelization of both, for example, by using similar items to increase the validity of the comparison. Although such efforts were made 20 years ago (e.g., Nosek, 2005) this issue has so far often been neglected by researchers in this field (Ren et al., 2025). Furthermore, some researchers (e.g., Peris et al., 2008; Stull et al., 2013) do not use already validated questionnaires to assess explicit mental illness stigma, which can also lead to a lack of validity. To address this, we first chose a validated German questionnaire to assess public mental illness stigma designed for samples with liberal values orientation (VASI; Rieckhof et al., 2021) and a validated self-stigma questionnaire (SSOSH; Zhou et al., 2019). The profession of psychotherapists requires an academic background and liberal orientations are more often represented in academic samples (Stubager, 2008). Second, we designed a SC-IAT (Karpinski and Steinman, 2006) using items from VASI and SSOSH to make the measurements more comparable, below, we describe this in detail.

#### 2.4.1 Explicit measures

Sociodemographic variables such as age, gender, highest degree of education (graduate academic subsample), personal experience with mental illness, experience with mental illness of important others, years of professional experience (psychotherapist subsample) and psychotherapy orientation (psychotherapist subsample) were assessed.

Public mental health stigma was assessed with the Value-based Stigma Inventory (VASI; Rieckhof et al., 2021). Participants are asked to rate the 15 items on a 5-point Likert scale from 1 = “strongly disagree” to 5 = “strongly agree.” Higher scores indicate higher public mental illness stigma. The items form five subscales: *Self-Realization, Personal Enrichment, Reputation, Meritocratic Values, Security*. Rieckhof et al. (2021) report good internal consistency (Cronbach’s *α* = 0.88), and good convergent and construct validity of their questionnaire.

Self-stigma of seeking help was assessed with the German version of the Self-Stigma of Seeking Help Scale (SSOSH; Zhou et al., 2019). Participants are asked to rate the 10 items on a 5-point Likert scale from 1 = “strongly disagree” to 5 = “strongly agree.” Higher scores indicate higher self-stigma of mental illness. Internal consistency for the scale is acceptable to good (0.80 ≤ *α* ≤ 0.84; Zhou et al., 2019).

Social desirability was assessed with KSE-G (Kemper et al., 2014). It consists of two subscales with three items each: *Exaggeration of Positive Qualities (PQ+)* and *Minimization of Negative Qualities (NQ*−*)*. The six items are rated on a 5-point scale, from 0 = “does not apply at all” to 4 = “applies completely.” Higher scores indicate lower social desirability for PQ+ and *higher* social desirability for NQ−, as the subscales are negatively correlated. Validation studies provide empirical evidence that the KSE-G offers not only a cost-effective but also a reliable (McDonald’s *ω* = 0.71 to 0.78) and valid assessment of socially desirable responding.

For convergent validity, we used the German version of the Social Distance Scale (SDS; Angermeyer et al., 2013), developed by Link et al. (1987). This scale includes various social scenarios of interactions with PLMI, such as renting a room, working together, being neighbors, caring for a young child, marrying into a family, introducing someone to friends, and recommending someone for a job. Participants use a 5-point Likert scale to indicate how willing or unwilling they would be to engage in each of these interactions with PLMI. Higher scores indicate lower wishes for social distance. The scale has been widely used in stigma research and shows good predictive validity and high internal consistency (Cronbach’s *α* = 0.91) (Butler and Gillis, 2011).

#### 2.4.2 Implicit measure

The SC-IAT was designed based on the items of VASI and SSOSH (see above). The final SC-IAT consisted of seven target items, rated on 11 pairs of evaluative items (see Table 1). Target items were, for example: Help from a psychotherapist, living with a mentally ill person and mentally ill family member. Positive vs. negative evaluative dimensions were e.g.: diligent vs. lazy, strong vs. weak and pleasant vs. unpleasant. The SC-IAT was programmed according to Karpinski and Steinman (2006), using the supplementary module “IAT” in SoSci Survey (Leiner, 2024) with the D-Score as an outcome variable of implicit attitudes. Higher D-Scores indicate lower implicit mental illness stigma. To ensure the internal validity of the implicit test, participants were asked to rate the subjective feasibility and distractibility during the task. We recorded the information about their handedness to control for differences between the groups.

Finally, at the end of the survey, we asked the participants whether they had any comments on the study. They could provide these in an open-ended response field or skip this question.

### 2.5 Statistical methods

The analyses were carried out on a group and individual level. The data was screened for outliers regarding the D-Score and VASI. Outliers were detected using the Tukey or “boxplot” method (Jones, 2019), outlier < (P25–2*iqr) or outlier > (P75 + 2*iqr), and were excluded from the analysis.

The statistical analysis was carried out using Jamovi 2.5 (The jamovi project, 2025). Post-hoc power analyses were conducted using G*Power 3.1 (Faul et al., 2009). *H1–H3* were examined using correlations. The internal consistency of the SC-IAT (*H1*) was assessed calculating the average correlation between the thirds and adjusting the value using the Spearman-Brown correction, resulting in adjusted *r* (Karpinski and Steinman, 2006; p. 19). *H4*–*H5* were examined using ANCOVAs instead of planned ANOVAs (as we had to control for age as a covariate, which we describe in the results section) and Tukey post-hoc tests.

The significance level was set to *α* = 0.05 for ANCOVA and Tukey post-hoc tests. Examining the data for significant differences between the subgroups, we used chi-square tests for dichotomous variables and ANOVA for age; the significance level of these tests was set to *α* = 0.01 to control for alpha-inflation. For tests for variance homogeneity (as part of the assumption tests) the significance level was set to *α* = 0.10.

The effect size for Tukey post-hoc-tests is Cohen’s *d*, for correlations the size of the (Pearson) correlation. According to Cohen (1988), |*d*| ≥ 0.20 and |*r*| ≥ 0.10 is considered a small, |*d*| ≥ 0.50 and |*r*| ≥ 0.30 a medium, and |*d*| ≥ 0.80 and |*r*| ≥ 0.50 a large effect size. For ANCOVA, the effect size is *ƞ*^2^; according to Cohen (1988) |*ƞ*^2^| ≥ 0.01 is considered a small, |*ƞ*^2^| ≥ 0.07 a medium, and |*ƞ*^2^| ≥ 0.14 a large effect size.

## 3 Results

In the laypeople sample, 21 people did not complete the questionnaire, and 15 did not complete it in the “psych” sample. Descriptive attrition analyses showed that the data entry process in both groups was interrupted either after reading and agreeing to the consent form or on the first pages after answering the sociodemographic questions or in the randomized condition “first implicit, then explicit” during the instructions for the implicit task. There were no significant differences regarding missing data between the randomized conditions. The final laypeople sample comprised n = 108 and the final “psych” sample *n* = 82 participants.

One D-Score outlier in the layperson sample and another one in the “psych” sample were excluded from analysis. Both were negative, suggesting higher stigmatizing attitudes more than four standard deviations away from the mean. Additional *N* = 27 (laypeople sample), respectively *N* = 21 (“psych” sample) participant D-Score datapoints were excluded due to too slow or too fast responses in accordance to the procedure used by Karpinski and Steinman (2006). Participants in the subsamples did not differ regarding the frequency of missing D-Score data. There were no significant differences in explicit value-based mental illness stigma measures between participants with complete D-Score measures and those with excluded D-Score measures. However, participants with excluded D-Score values had significantly lower values in SDS questionnaire with a small effect size: [*t* (188) = −2.23, *p* = 0.027, *d* = −0.37], which means that participants with greater desire for social distance were less likely to complete the implicit task.

In the “psych” study, 30 participants provided comments on the study (60% psychotherapists, 40% psychology students). In the laypeople subsample, 37 participants left comments (59% graduated academics, 41% non-psychology students). The comments referred to the general feasibility of the study or to a differentiate view on mental illness, which participants did not feel represented in our questions, like: “Psychiatric disorders are very diverse, so answering in general was sometimes difficult. I think my attitudes toward whether I would leave a child with a person with depression or anxiety disorder versus with a person, for example, with schizophrenia, would be quite different.” Such responses were more often noted by psychotherapists rather than participants from the other subgroups.

### 3.1 Descriptive statistics

In the laypersons sample, 81.5% of the non-psychological student population were undergraduate students. 18.5% were Master students or comparable (“Staatsexamen”). In the sample with completed academic education (“graduated academics”) 60.7% held a degree similar to master or diploma, 30.4% held a Ph.D. and the rest held other university degrees (e.g., “Staatsexamen”).

In the “psych” sample, all students were undergraduate psychology students.

In the subsample of psychotherapist (trainees), the psychotherapy approaches most frequently represented were cognitive behavioral psychotherapy (*n* = 21), followed by psychodynamic (*n* = 11) and systemic psychotherapy (*n* = 8). The subsample consisted of 22 psychotherapist trainees, 12 of them with more than 2 years of training. Out of the 18 licensed psychotherapists, 15 had more than 2 years of professional experience. Years of experience showed small (0.10 ≥ |*r*| ≤ 0.30) non-significant correlations with explicit stigma measures and with the D-score, respectively. However, the direction of the correlations suggested a positive effect of experience on lower levels of implicit and explicit stigma. The participant characteristics are shown in Supplementary Table S1.

No significant differences in subsamples could be detected regarding handedness (left/right), the level of experience with mental illness of others, frequency of personal experiences with mental illness, subjective feasibility and the distractibility during the task and gender. As the difference in age was highly significant between the groups [*F* (3, 186) = 101, *p* < 0.001], it was included as a covariate.

### 3.2 Results for the main hypotheses

In the total sample, the SC-IAT showed high internal consistency (adjusted *r* = 0.87), in line with our hypothesis (*H1*). The explicit scales showed mostly satisfactory or good internal consistency: VASI Cronbach’s *α* = 0.75, SSOSH Cronbach’s *α* = 0.71, SDS Cronbach’s *α* = 0.86. The reliability of the KSE-G subscales is acceptable (PQ+ McDonald’s *ω* = 0.67, respectively NQ− McDonald’s *ω* = 0.66).

To test *H2*, we explored the correlations of SC-IAT and VASI, SSOSH, SDS, and the KSE-G subscales (see Table 2). The correlations between D-Score and VASI were negative, small and significant (1-*β* = 0.78), supporting *H2*. Yet, contrary to our prediction, there was no correlation between D-Score and SSOSH (1-*β* = 0.87). In contrast to our hypothesis, there was also a small but significant positive correlation between D-Score and SDS (1-*β* = 0.76), which indicates that lower implicit stigma values are accompanied by a lower desire for social distance with PLMI.

**Table 2:** Pearson correlations between explicit outcomes and implicit SC-IAT D-Score.

	VASI	SSOSH	SDS	KSE-G PQ+	KSE-G PQ−
SSOSH	0.293*** df = 186 *p* < 0.001				
SDS	−0.566*** df = 187 *p* < 0.001	−0.316*** df = 187 *p* < 0.001			
KSE-G PQ+	−0.129 df = 187 *p* = 0.078	−0.272*** df = 187 *p* < 0.001	0.114 df = 188 *p* = 0.117		
KSE-G PQ−	−0.041 df = 187 *p* = 0.576	0.144* df = 187 *p* = 0.049	−0.023 df = 188 *p* = 0.752	−0.379*** df = 188 *p* < 0.001	
D-Score	−0.202* df = 138 *p* = 0.017	−0.020 df = 138 *p* = 0.818	0.196* df = 138 *p* = 0.021	0.073 df = 138 *p* = 0.392	−0.237** df = 138 *p* = 0.005

VASI, Value-Based Stigma Inventory; SSOSH, Self-Stigma of Seeking Help questionnaire; SDS, Social Distance Scale; KSE-G PQ+, Social Desirability questionnaire “Kurzskala Soziale Erwünschtheit-Gamma”, subscale maximisation of positive qualities; KSE-G NQ−, Social Desirability questionnaire “Kurzskala Soziale Erwünschtheit-Gamma”, subscale minimisation of negative qualities; D-Score, outcome of Single Category Implicit Association Task (SC-IAT).

*p < 0.05. **p < 0.01. ***p < 0.001.

Regarding *H3*, contrary to our prediction, the SC-IAT showed small negative significant correlations with the social desirability subscale NQ− of the KSE-G (1-*β* = 0.70). This indicates that lower implicit stigma is related to lower social desirability concerning the minimization of negative personal characteristics.

To test *H4*, an ANCOVA for VASI stigma scores pointed to a significant difference between the groups (see Table 3). Post-hoc tests revealed a significant difference between the group of graduated academics and the psychotherapist (trainee) group with a medium size effect. These results partially support our hypothesis, as only the subgroup of psychotherapists and graduate academics significantly differed, but not the subgroup of laypeople students and psychology students.

**Table 3:** The results of ANCOVA and post-hoc tests on explicit VASI mental illness stigma across the subsamples.

	Sum of squares	df	Mean square deviation	*F*	*p*	*η* ^2^
Age	5.64	1	5.64	4.19	0.042*	0.02
Subsample	13.13	3	4.38	3.25	0.023*	0.05
Residual	247.69	184	1.35			

Post-hoc tests						
Subsamples	M	SD	df	*t*	*p*_Tukey_	Cohen’s *d*
4–3	0.17	0.34	184	0.501	0.96	0.15
4–2	0.72	0.27	184	2.679	0.04*	**0.62**
4–1	0.11	0.34	184	0.338	0.99	0.10
3–2	0.55	0.28	184	1.955	0.21	0.47
3–1	−0.06	0.24	184	−0.236	0.99	−0.05
2–1	−0.61	0.28	184	−2.138	0.15	−0.52

Comparisons are based on estimated marginal means. M, mean; SD, standard deviation; subsamples: 1, psychology students; 2, psychotherapists; 3, layperson students; 4, graduate academic laypersons.

*p < 0.05, bold values highlight significant differences between subsamples.

Regarding *H5*, in contrast to our hypothesis, no significant differences were found in implicit SC-IAT D-Scores between the subsamples: *F* (3, 135) = 0.171, *p* = 0.916, 1-*β* = 0.93, thereby rejecting *H5*.

## 4 Discussion

The purpose of this study was to examine implicit and explicit stigmatizing attitudes toward mental illness in a psychotherapist sample in contrast to psychology students and academic laypersons. We created an implicit task based on the explicit questionnaires regarding public stigma (VASI) and self-stigma (SSOSH) in order to enhance validity and reliability of the implicit SC-IAT task. There was no significant difference between psychology students and psychotherapists regarding explicit mental illness stigma measured with VASI. But VASI values of psychotherapists and graduate academic laypersons differed with a medium effect size, last ones showing higher mental illness stigma. Concerning implicit mental illness stigma, no significant group differences could be detected.

The results show high internal consistency for the SC-IAT in our sample, which suggests that developing an implicit stigma test based on explicit stigma items represents a valid procedure (*H1*). It is likely that this parallelization procedure led to small and significant correlations in the predicted direction with VASI; however, no correlation with SSOSH was found (*H2*). This may be because only few SSOSH items were used.

### 4.1 Implicit measure and social desirability

Contrary to the prediction, the absolute values of the positive significant correlation of D-Score and SDS are similar to the correlation size of D-Score and VASI (*H2*). So, despite our parallelization of the implicit task with the explicit VASI questionnaire as a measure for mental illness stigma in liberal cohorts, the implicit SC-IAT scores also seem to reflect the desire for social distance. As excluded D-Score measures and SDS were connected in our sample, this connection may reflect a more behavior-based stigma measure in contrast to questionnaires. Moreover, D-Score items show a small and significant correlation with the KSE-G Minimization of Negative Qualities (NQ−) subscale. At this point, questions might arise concerning the validity of an implicit task like SC-IAT being a reliable implicit stigma measure, which is not new in this research area (Gawronski and Conrey, 2006; Greenwald and Banaji, 2017). We addressed this issue by parallelizing explicit and implicit measures and assessing social desirability. Karpinski and Steinman (2006) argue that the SC-IAT is not susceptible to social desirability bias, as people whose response times are too slow or too fast are excluded from the analyses (p. 19). However, while the implicit scores show a small and significant correlation with the social desirability subscale, measuring a moralistic bias by minimizing one’s negative characteristics, the explicit ones do not correlate with the KSE-G subscales. Such differences in socially desirable response behavior have been found for explicit measures, but not for implicit ones (Anderson, 2019). An explanation might be that the significant negative intercorrelation between the KSE-G NQ− scale and the D-score may indicate that a lower tendency to minimize one’s own negative qualities is associated with more candid responding in the implicit task. This, however, raises the question of why the KSE-G PQ+ scale does not exhibit comparable correlations with the D-score. An alternative explanation may lie in differences in cognitive processing styles, which could have allowed participants either to deliberate longer on certain aspects of the study questions or to respond more quickly. Neither overall completion time nor the time spent on individual questionnaire pages was significantly associated with implicit or explicit outcome measures. Our contrasting results, can be due to our choice of KSE-G as a social desirability questionnaire: Other studies (Anderson, 2019; Danioni et al., 2020) used the Balanced Inventory of Desirable Responding (BDIR) created by Paulhus (e.g., Hart et al., 2015). When creating KSE-G, Kemper et al. focused on communal values only, aiming to capture the moralistic bias in socially desirable responding (Kemper et al., 2014). They found no correlation between BIDR and KSE-G subscales but medium to high correlations between KSE-G subscales and the Big 5 factor Conscientiousness, respectively, the Minnesota Multiphasic Personality Inventory-2 (MMPI-2) lie subscale. Thus, the KSE-G may not constitute a reliable measure of socially desirable responding, but rather of social desirability as a personality trait. Steiger et al. (2022) identified an association between specific personality traits and explicit public stigma toward mental illness. As there are small but significant correlations with KSE-G in our sample, it might be that implicit task measures reflect not only attitudes towards mentally ill people, but also other relevant psychological constructs such as personality traits or personal values. As personality traits and personal values can be connected to attitudes (Boer and Fischer, 2013), a correlation between such constructs seems reasonable.

### 4.2 Dissonance between implicit and explicit mental illness stigma in psychotherapists

We found significant differences regarding stigmatizing attitudes between the subgroups (*H4*). We expected a significant difference in the sample regarding explicit stigmatizing attitudes between psychology students and psychotherapists, but no such difference could be detected (*H4a*). But there was a significant difference regarding explicit stigmatizing attitudes between the subsample of graduate academic laypersons and the psychotherapist subsample, the latter showing the lowest values of explicit stigmatizing attitudes regarding mental illness (*H4b*). One could argue, that this could be related to the graduate academic sample having the least experience with PLMI (Hackler et al., 2016) compared to the other samples. But it does not explain why there are no significant differences between both student samples with high levels of experience with PLMI and the sample of graduated academics. Summarized, the significant medium sized difference cannot be solely explained by higher familiarity with mental illness, by social desirability effects or by years of professional experience of psychotherapists. Specific aspects of psychotherapists’ training or professional values seem more likely to be the explanation. Maybe it leads to a more differentiated view on mental illness (as mirrored in the open questions responses) and therefore a more profound understanding of mental illness being a continuum rather than a dichotomous state, a belief, which is connected to lower mental illness stigma (Peter et al., 2021; Tomczyk et al., 2023).

Regarding implicit attitudes measured by SC-IAT D-Score, no differences could be detected (*H5*), contrary to our prediction as we expected differences between the “psych” and layperson subsamples. As the *post hoc* power for this null finding was strong, it is unlikely that a meaningful effect was missed. Although three of our subsamples showed low levels of explicit mental illness stigma and high levels of contact with PLMI, as well as personal experience with mental illness, implicit mental illness stigma was slightly negatively biased, as reflected by the negative D-score values (see Supplementary Table S1). This could reflect a negative view of mental illness which is deeply embedded in society (e.g., Ren et al., 2025) and is only changing at a slow pace.

The Dual Attitude Model (Wilson et al., 2000) assumes, that implicit and explicit attitudes towards the same object can co-exist, although they have different valences. So, people can have more than one evaluation of the same subject, the explicit one requires more cognitive or time resources and the implicit one can be retrieved more automatically from memory (Wilson et al., 2000). Consequently, it could be that over the course of several years of psychotherapy training and in-depth self-experience, *some* psychotherapists learn to reflect more critically upon their own negative assumptions about PLMI. This learning could result in lower explicit mental illness stigma, even if negative implicit assumptions remain as negative as in other populations. On the other hand, “implicit attitudes and stereotypes may be acquired over many years from language and social experiences” and “may be as difficult or impossible to unlearn as are musical, medical, and athletic expertise” (Greenwald and Banaji, 2017; pp. 866ff).

Current research also indicates that implicit attitudes can be changed (Dasgupta, 2013; Kurdi et al., 2025), but this change occurs due to changes in “local environments” (e.g., Dasgupta, 2013, p. 239ff) and potentially via “social-group specific mechanisms”(Kurdi et al., 2025 p. 1661). How might this translate to the field of psychotherapeutic training? Longitudinal study results (Kurdi et al., 2025) suggest that changes in explicit and implicit attitudes may influence each other, but not necessarily. Therefore, at this point and from our perspective, no differential recommendations can be made regarding the modification of explicit versus implicit attitudes. It remains to be investigated how and with what intensity certain interventions for psychotherapists could influence the change of implicit versus explicit stigmatizing attitudes. At present, we can only speculate about how such interventions may be effective in reducing implicit bias among psychotherapists, as, to the best of our knowledge, no empirical studies have directly addressed this issue. Qualitative methods could help to identify which factors may contribute to psychotherapists’ potentially lower levels of explicit stigma, or whether and in which domains they nevertheless exhibit stigma toward mental illness. Psychotherapists’ increased awareness of their own stigmatizing tendencies and of the potential risks associated with such biases may in itself constitute a first step toward change and it potentially could change the “local environment” cited above. Additionally, it may be beneficial to promote a recovery-oriented perspective among psychotherapists more broadly (O’Connor and Yanos, 2023), for example by providing opportunities during and after professional training to engage with PLMI who have achieved a satisfying and meaningful life or by focusing on the mental illness and mental health continuum rather than focusing solely on diagnostic categories (despite their clear relevance for professional practice). Furthermore, given that the induction of cognitive dissonance has been shown to facilitate changes in stigmatizing attitudes (Meaney and Rieger, 2021), interventions explicitly targeting cognitive dissonance regarding stereotypes of PLMI hold by psychotherapists may therefore be a promising approach To evaluate such interventions, outcomes like clinical decision making (Peris et al., 2008) or changes in attitudes towards highly stigmatized patient groups (O’Connor and Yanos, 2023) might be potentially relevant outcome measures.

### 4.3 Limitations

Excluded D-Scores were connected to higher social distance values (and thus greater desire for social distance with PLMI), so this limits the generalization of results. As non-completers were significantly more likely to be assigned to the condition in which the implicit task was presented first, this may also have been the case for them. Performing the implicit task might be a greater or minor challenge for participants according to their desire for social distance. To our knowledge, this effect has not yet been reported in the literature on implicit mental illness stigma, and it may be worth examining in greater depth, as it points to more behavior-based aspects of measuring mental illness stigma. Furthermore, with exception of the academic laypersons’ subsample, participants in our sample were very familiar with mental illness issues, either due to lived experience or because they knew PLMI, so the findings could be specific to our studied population.

Only in the subsample of graduate academics, there was a similar proportion of female and male participants. In other subsamples, participants with female gender were predominant, which may reflect the reality, as there are more psychotherapists who define themselves as female (BPtK, 2016).

As stigma varies across diagnoses (e.g., Schomerus et al., 2023) we cannot rule out that the observed implicit bias may be driven primarily by associations with more highly stigmatized conditions. Schizophrenia and substance use disorders are more likely to be stigmatized than depression (e.g., Schomerus et al., 2023), and this effect has been shown for psychotherapists, too (Gonzales et al., 2021; O’Connor and Yanos, 2023).

## 5 Conclusion

Psychotherapists showed the lowest explicit stigmatizing attitudes but still held negatively biased implicit stigmatizing attitudes. As implicit mental illness stigma can be connected to restrictive practices, e.g., medication (Stull et al., 2013) and over-diagnosis (Peris et al., 2008), it seems important to further examine implicit mental illness stigma in psychotherapists. There is evidence that self-stigma and public mental illness stigma of psychotherapy patients is connected to working alliance and therapeutic engagement and that both types of stigma can decrease over the course of psychotherapy treatment (Deres et al., 2020; Kendra et al., 2014). But nothing is known yet about the role of mental illness stigma of the psychotherapist in this process. Negative implicit bias may influence psychotherapeutic practice in multiple ways, potentially undermining the therapeutic alliance, perceived empathic engagement, treatment intensity, and clinicians’ comfort in addressing certain topics. Psychotherapists should be aware of their own implicit tendency to stigmatize mental illness and its potential causes (like over-diagnosis) on their treatment practices. This knowledge could be part of psychotherapist training curriculum and supervision, e.g., in fostering recovery-oriented views on mental illness.

Furthermore, future studies should try to examine how psychotherapists’ training could have an impact on implicit attitudes of psychotherapists towards PLMI in reducing or enhancing mental illness stigma.

Beyond this, future research should focus on developing diagnosis-specific implicit measurement methods to more clearly identify potential negative bias.

Finally, implicit and explicit mental illness stigma of psychotherapists should be further examined in regard to personal values and continuum beliefs.

## Data Availability

The raw data supporting the conclusions of this article will be made available by the authors, without undue reservation.

## References

[ref1] Anderson J. R. (2019). The moderating role of socially desirable responding in implicit–explicit attitudes toward asylum seekers. Int. J. Psychol. 54, 1–7. doi: 10.1002/ijop.12439, 28675437

[ref2] Angermeyer M. C. Matschinger H. Schomerus G. (2013). Attitudes towards psychiatric treatment and people with mental illness: changes over two decades. Br. J. Psychiatry 203, 146–151. doi: 10.1192/bjp.bp.112.122978, 23787060

[ref3] Ärzteblatt, D.Ä.G., Redaktion Deutsches (2011a). Psychotherapeutische Versorgung: Auch Ärzte machen Psychotherapie [WWW Document]. Dtsch. Ärztebl. Available online at: https://www.aerzteblatt.de/archiv/psychotherapeutische-versorgung-auch-aerzte-machen-psychotherapie-f5295116-13a4-4f8f-b1df-30ee466feabf (Accessed July 31, 2025).

[ref4] Ärzteblatt, D.Ä.G., Redaktion Deutsches (2011b). Psychotherapie in Polen: Viele ungelöste Probleme [WWW Document]. Dtsch. Ärztebl. Available online at: https://www.aerzteblatt.de/archiv/psychotherapie-in-polen-viele-ungeloeste-probleme-bd71e74e-7dcb-42e2-a9cf-bd0c6b46fe13 (Accessed May 19, 2025).

[ref5] Boer D. Fischer R. (2013). How and when do personal values guide our attitudes and sociality? Explaining cross-cultural variability in attitude-value linkages. Psychol. Bull. 139, 1113–1147. doi: 10.1037/a0031347, 23339521

[ref6] BPtK, (2016). Das Heft in die eigene Hand nehmen [WWW Document]. Available online at: https://www.bptk.de/neuigkeiten/das-heft-in-die-eigene-hand-nehmen (Accessed August 27, 2025).

[ref7] Butler R. C. Gillis J. M. (2011). The impact of labels and behaviors on the stigmatization of adults with Asperger’s disorder. J. Autism Dev. Disord. 41, 741–749. doi: 10.1007/s10803-010-1093-9, 20811769

[ref8] Clement S. Schauman O. Graham T. Maggioni F. Evans-Lacko S. Bezborodovs N. . (2015). What is the impact of mental health-related stigma on help-seeking? A systematic review of quantitative and qualitative studies. Psychol. Med. 45, 11–27. doi: 10.1017/S0033291714000129, 24569086

[ref9001] Cohen J. (1988). Statistical power analysis for the behavioral sciences (2nd ed.) Lawrence Erlbaum.

[ref9] Danioni F. Coen S. Rosnati R. Barni D. (2020). The relationship between direct and indirect measures of values: is social desirability a significant moderator? Eur. Rev. Appl. Psychol. 70:100524. doi: 10.1016/j.erap.2020.100524

[ref10] Dasgupta N. (2013). “Implicit attitudes and beliefs adapt to situations: a decade of research on the malleability of implicit prejudice, stereotypes, and the self-concept” in Advances in experimental social psychology (Academic Press), 233–279.

[ref11] De Cuyper K. De Houwer J. Vansteelandt K. Perugini M. Pieters G. Claes L. . (2017). Using indirect measurement tasks to assess the self-concept of personality: a systematic review and Meta-analyses. Eur. J. Personal. 31, 8–41. doi: 10.1002/per.2092

[ref12] Del Olmo-Romero F. González-Blanco M. Sarró S. Grácio J. Martín-Carrasco M. Martinez-Cabezón A. C. . (2019). Mental health professionals’ attitudes towards mental illness: professional and cultural factors in the INTER NOS study. Eur. Arch. Psychiatry Clin. Neurosci. 269, 325–339. doi: 10.1007/s00406-018-0867-5, 29353369

[ref13] Deres A. T. Bürkner P.-C. Klauke B. Buhlmann U. (2020). The role of stigma during the course of inpatient psychotherapeutic treatment in a German sample. Clin. Psychol. Psychother. 27, 239–248. doi: 10.1002/cpp.2423, 31910308

[ref14] Faul F. Erdfelder E. Buchner A. Lang A.-G. (2009). Statistical power analyses using G*power 3.1: tests for correlation and regression analyses. Behav. Res. Methods 41, 1149–1160. doi: 10.3758/BRM.41.4.1149, 19897823

[ref15] Gallimore J.-B. Diaz K. G. Gunasinghe C. Thornicroft G. Salisbury T. T. Gronholm P. C. (2023). Impact of mental health stigma on help-seeking in the Caribbean: systematic review. PLoS One 18:e0291307. doi: 10.1371/journal.pone.0291307, 37699044 PMC10497129

[ref16] Gawronski B. Conrey F. R. (2006). Der Implizite Assoziationstest als Maß automatisch aktivierter Assoziationen: Reichweite und Grenzen. Psychol. Rundsch.

[ref17] Girma E. Tesfaye M. Froeschl G. Möller-Leimkühler A. Müller N. Dehning S. (2013). Public stigma against people with mental illness in the Gilgel gibe field research center (GGFRC) in Southwest Ethiopia. PLoS One 8:e82116. doi: 10.1371/journal.pone.0082116, 24324756 PMC3853185

[ref18] Gonzales L. López-Aybar L. McCullough B. (2021). Variation in provider attitudes and treatment recommendations for individuals with schizophrenia and additional marginalized identities: a mixed-method study. Psychiatr. Rehabil. J. 44, 107–117. doi: 10.1037/prj0000461, 33600201

[ref19] Greenwald A. G. Banaji M. R. (2017). The implicit revolution: reconceiving the relation between conscious and unconscious. Am. Psychol. 72, 861–871. doi: 10.1037/amp0000238, 29283625

[ref20] Greenwald A. G. McGhee D. E. Schwartz J. L. K. (1998). Measuring individual differences in implicit cognition: the implicit association test. J. Pers. Soc. Psychol. 74, 1464–1480. doi: 10.1037/0022-3514.74.6.1464, 9654756

[ref21] Hackler A. H. Cornish M. A. Vogel D. L. (2016). Reducing mental illness stigma: effectiveness of hearing about the normative experiences of others. Stigma Health 1, 201–205. doi: 10.1037/sah0000028

[ref22] Hart C. M. Ritchie T. D. Hepper E. G. Gebauer J. E. (2015). The balanced inventory of desirable responding short form (BIDR-16). SAGE Open 5:2158244015621113. doi: 10.1177/2158244015621113

[ref23] Hazell C. M. Berry C. Bogen-Johnston L. Banerjee M. (2022). Creating a hierarchy of mental health stigma: testing the effect of psychiatric diagnosis on stigma. BJPsych Open 8:e174. doi: 10.1192/bjo.2022.578, 36156196 PMC9534883

[ref24] Jacobi F. Höfler M. Siegert J. Mack S. Gerschler A. Scholl L. . (2014). Twelve-month prevalence, comorbidity and correlates of mental disorders in Germany: the mental health module of the German health interview and examination survey for adults (DEGS1-MH). Int. J. Methods Psychiatr. Res. 23, 304–319. doi: 10.1002/mpr.1439, 24729411 PMC6878234

[ref25] Jacobi F. Höfler M. Strehle J. Mack S. Gerschler A. Scholl L. . (2015). Twelve-months prevalence of mental disorders in the German health interview and examination survey for adults – mental health module (DEGS1-MH): a methodological addendum and correction. Int. J. Methods Psychiatr. Res. 24, 305–313. doi: 10.1002/mpr.1479, 26184561 PMC6878329

[ref26] James J. S. (2018). Explicit, implicit, and behavioral stigmatization of mental illness. Southern Mississippi: University of Southern Mississippi.

[ref27] Jones P. R. (2019). A note on detecting statistical outliers in psychophysical data. Atten. Percept. Psychophys. 81, 1189–1196. doi: 10.3758/s13414-019-01726-3, 31089976 PMC6647454

[ref28] Karpinski A. Steinman R. B. (2006). The single category implicit association test as a measure of implicit social cognition. J. Pers. Soc. Psychol. 91, 16–32. doi: 10.1037/0022-3514.91.1.16, 16834477

[ref29] Kemper C.J. Beierlein C. Bensch D. Kovaleva A. Rammstedt B. (2014). Soziale Erwünschtheit-Gamma (KSE-G). Zusammenstellung Sozialwissenschaftlicher Items Skalen ZIS. doi: 10.6102/zis186

[ref30] Kendra M. S. Mohr J. J. Pollard J. W. (2014). The stigma of having psychological problems: relations with engagement, working alliance, and depression in psychotherapy. Psychotherapy 51, 563–573. doi: 10.1037/a0036586, 24866971

[ref31] Kim L.S. Nainee S. Viapude G.N. Aun T.S. (2019). Interactional effect of educational level and gender on public stigmatization. doi: 10.15405/epsbs.2019.09.2

[ref32] Kopera M. Suszek H. Bonar E. Myszka M. Gmaj B. Ilgen M. . (2015). Evaluating explicit and implicit stigma of mental illness in mental health professionals and medical students. Community Ment. Health J. 51, 628–634. doi: 10.1007/s10597-014-9796-6, 25535045 PMC4475542

[ref33] Kurdi B. Charlesworth T. E. S. Mair P. (2025). International stability and change in explicit and implicit attitudes: an investigation spanning 33 countries, five social groups, and 11 years (2009–2019). J. Exp. Psychol. Gen. 154, 1643–1666. doi: 10.1037/xge0001746, 40167532

[ref34] Lauber C. Nordt C. Braunschweig C. Rössler W. (2006). Do mental health professionals stigmatize their patients? Acta Psychiatr. Scand. 113, 51–59. doi: 10.1111/j.1600-0447.2005.00718.x, 16445483

[ref35] Leiner D.J. (2024). SoSci survey (Version 3.5.02) [Computer software]. Available online at: https://www.soscisurvey.de

[ref36] Link B. G. Cullen F. T. Frank J. Wozniak J. F. (1987). The social rejection of former mental patients: understanding why labels matter. Am. J. Sociol. 92, 1461–1500. doi: 10.1086/228672

[ref37] Link B. G. Phelan J. C. (2001). Conceptualizing stigma. Annu. Rev. Sociol. 27, 363–385. doi: 10.1146/annurev.soc.27.1.363

[ref38] Lo L. L. H. Suen Y. N. Chan S. K. W. Sum M. Y. Charlton C. Hui C. L. M. . (2021). Sociodemographic correlates of public stigma about mental illness: a population study on Hong Kong’s Chinese population. BMC Psychiatry 21:274. doi: 10.1186/s12888-021-03301-3, 34051783 PMC8164229

[ref39] López-Aybar L. Gonzales L. (2024). Prosumers’ experiences of witnessed discrimination and internalized stigma: a moderated mediation. Psychol. Serv. 21, 982–992. doi: 10.1037/ser0000837, 38358700

[ref40] López-Aybar L. Gonzales L. Kanani A. (2024). Prosumers’ experiences of stigma dimensions within the clinical psychology field. Psychol. Serv. 21, 369–378. doi: 10.1037/ser0000765, 37023291

[ref41] Mack S. Jacobi F. Gerschler A. Strehle J. Höfler M. Busch M. A. . (2014). Self-reported utilization of mental health services in the adult German population--evidence for unmet needs? Results of the DEGS1-mental health module (DEGS1-MH). Int. J. Methods Psychiatr. Res. 23, 289–303. doi: 10.1002/mpr.1438, 24687693 PMC6878535

[ref42] McLaren T. Peter L.-J. Tomczyk S. Muehlan H. Schomerus G. Schmidt S. (2023). The seeking mental health care model: prediction of help-seeking for depressive symptoms by stigma and mental illness representations. BMC Public Health 23:69. doi: 10.1186/s12889-022-14937-5, 36627597 PMC9831378

[ref43] Meaney T. Rieger E. (2021). Integrating cognitive dissonance and social consensus to reduce weight stigma. Body Image 37, 117–126. doi: 10.1016/j.bodyim.2021.02.003, 33647827

[ref44] Nosek B. A. (2005). Moderators of the relationship between implicit and explicit evaluation. J. Exp. Psychol. Gen. 134, 565–584. doi: 10.1037/0096-3445.134.4.565, 16316292 PMC1440676

[ref45] Nosek B. A. Banaji M. R. (2001). The go/no-go association task. Soc. Cogn. 19, 625–666. doi: 10.1521/soco.19.6.625.20886

[ref46] O’Connor L. K. Yanos P. (2023). Training and individual correlates of attitudes toward serious mental illness among clinical psychology doctoral students. Train. Educ. Prof. Psychol. 17, 295–303. doi: 10.1037/tep0000422

[ref47] O’Connor L. K. Yanos P. T. (2021). Where are all the psychologists? A review of factors impacting the underrepresentation of psychology in work with serious mental illness. Clin. Psychol. Rev. 86:102026. doi: 10.1016/j.cpr.2021.102026, 33813162

[ref48] Peris T. S. Teachman B. A. Nosek B. A. (2008). Implicit and explicit stigma of mental illness: links to clinical care. J. Nerv. Ment. Dis. 196, 752–760. doi: 10.1097/NMD.0b013e3181879dfd, 18852619

[ref49] Peter L.-J. Schindler S. Sander C. Schmidt S. Muehlan H. McLaren T. . (2021). Continuum beliefs and mental illness stigma: a systematic review and meta-analysis of correlation and intervention studies. Psychol. Med. 51, 716–726. doi: 10.1017/S0033291721000854, 33827725 PMC8108391

[ref50] Ren Y. Wang S. Fu X. Shi X. (2025). A systematic review and Meta-analysis of implicit stigma toward people with mental illness among different groups: measurement, extent, and correlates. Psychol. Res. Behav. Manag. 18, 851–875. doi: 10.2147/PRBM.S503942, 40226437 PMC11989592

[ref51] Rieckhof S. Sander C. Speerforck S. Prestin E. Angermeyer M. C. Schomerus G. (2021). Development and validity of the value-based stigma inventory (VASI): a value-sensitive questionnaire for the assessment of mental health stigma. BMC Psychiatry 21:570. doi: 10.1186/s12888-021-03427-4, 34781933 PMC8594194

[ref52] Sandhu H. S. Arora A. Brasch J. Streiner D. L. (2019). Mental health stigma: explicit and implicit attitudes of Canadian undergraduate students, medical school students, and psychiatrists. Can. J. Psychiatry 64, 209–217. doi: 10.1177/0706743718792193, 30058372 PMC6405810

[ref53] Schomerus G. Lüders J. (2023). “Stigmatisierung und Psychotherapie – Wie betrachten Psychotherapeuten sich selbst und andere?” in Psychotherapeuten und das Altern: Die Bedeutung des Alterns in der therapeutischen Beziehung und der eigenen Lebensgeschichte. eds. Strauß B. Spitzer C. (Berlin/Heidelberg: Springer), 141–151. doi: 10.1007/978-3-662-65228-2_11

[ref54] Schomerus G. Spahlholz J. Speerforck S. (2023). Die Einstellung der deutschen Bevölkerung zu psychischen Störungen. Bundesgesundheitsbl. Gesundheitsforsch. Gesundheitsschutz 66, 416–422. doi: 10.1007/s00103-023-03679-3, 36853345 PMC9972325

[ref55] Schomerus G. Stolzenburg S. Freitag S. Speerforck S. Janowitz D. Evans-Lacko S. . (2019). Stigma as a barrier to recognizing personal mental illness and seeking help: a prospective study among untreated persons with mental illness. Eur. Arch. Psychiatry Clin. Neurosci. 269, 469–479. doi: 10.1007/s00406-018-0896-0, 29679153

[ref56] Seewald A. Teige-Mocigemba S. Rief W. (2023). Outcome expectations in psychotherapy: validation of the therapy single category implicit association test (therapy sc-iat). Cogn. Ther. Res. 47. doi: 10.1007/s10608-023-10413-5

[ref57] Statistisches Bundesamt (2021). Zahl der Psychotherapeutinnen und -therapeuten von 2015 bis 2019 um 19% gestiegen [WWW Document]. Stat. Bundesamt. Available online at: https://www.destatis.de/DE/Presse/Pressemitteilungen/2021/03/PD21_N022_23.html (Accessed July 31, 2025).

[ref58] Steiger S. Sowislo J. F. Moeller J. Lieb R. Lang U. E. Huber C. G. (2022). Personality, self-esteem, familiarity, and mental health stigmatization: a cross-sectional vignette-based study. Sci. Rep. 12:10347. doi: 10.1038/s41598-022-14017-z, 35725744 PMC9209478

[ref59] Stubager R. (2008). Education effects on authoritarian–libertarian values: a question of socialization1. Br. J. Sociol. 59, 327–350. doi: 10.1111/j.1468-4446.2008.00196.x, 18498598

[ref60] Stull L. G. McGrew J. H. Salyers M. P. Ashburn-Nardo L. (2013). Implicit and explicit stigma of mental illness: attitudes in an evidence-based practice. J. Nerv. Ment. Dis. 201, 1072–1079. doi: 10.1097/NMD.0000000000000056, 24284643 PMC4031039

[ref61] The jamovi project (2025). Jamovi (Version 2.6) [Computer Software]. Available online at: https://www.jamovi.org

[ref62] Thornicroft G. Rose D. Kassam A. (2007). Discrimination in health care against people with mental illness. Int. Rev. Psychiatry 19, 113–122. doi: 10.1080/09540260701278937, 17464789

[ref63] Tomczyk S. Schlick S. Gansler T. McLaren T. Muehlan H. Peter L.-J. . (2023). Continuum beliefs of mental illness: a systematic review of measures. Soc. Psychiatry Psychiatr. Epidemiol. 58, 1–16. doi: 10.1007/s00127-022-02345-4, 35927343 PMC9845169

[ref64] Ungar T. Knaak S. Szeto A. C. (2016). Theoretical and practical considerations for combating mental illness stigma in health care. Community Ment. Health J. 52, 262–271. doi: 10.1007/s10597-015-9910-4, 26173403 PMC4805707

[ref65] Wang P.-W. Ko C.-H. Chen C.-S. Yang Y.-H. C. Lin H.-C. Cheng C.-C. . (2016). Changes of explicit and implicit stigma in medical students during psychiatric clerkship. Acad. Psychiatry 40, 224–228. doi: 10.1007/s40596-015-0432-8, 26449982

[ref66] Williams B. J. Kaufmann L. M. (2012). Reliability of the go/no go association task. J. Exp. Soc. Psychol. 48, 879–891. doi: 10.1016/j.jesp.2012.03.001

[ref67] Wilson T. D. Lindsey S. Schooler T. Y. (2000). A model of dual attitudes. Psychol. Rev. 107, 101–126. doi: 10.1037/0033-295X.107.1.101, 10687404

[ref68] Zhou Y. Lemmer G. Xu J. Rief W. (2019). Cross-cultural measurement invariance of scales assessing stigma and attitude to seeking professional psychological help. Front. Psychol. 10:1249. doi: 10.3389/fpsyg.2019.01249, 31214074 PMC6554279

