# Supplementary material for: Blind spots of psychotherapists? Implicit and explicit mental illness stigma in psychotherapists, psychology students, and laypersons
Source: Front Psychol. 2026 Feb 27;17:1759801. doi: 10.3389/fpsyg.2026.1759801 (PMC12983224; doi:10.3389/fpsyg.2026.1759801)
Supplement: Supplementary file 1 [file Table_1.docx]

|  | **„Laypeople“ Sample** | | **„Psych“ Sample** | |
| --- | --- | --- | --- | --- |
|  | **Students** | **Graduate Academics** | **Psychology students** | **Psychotherapists (in training)** |
| **Sample size**  **(Sample size D-Score)** | N = 53  (N = 41) | N = 55  (N = 39) | N = 42  (N = 33) | N = 40  (N = 27) |
| **Age** **(in percent)**  18-24ys  25-34ys  35-44ys  45-54ys  55-64ys  ≥ 65ys | 81.5%  18.5%  0  0  0  0 | 0  20%  14.5%  18.2%  30.9%  16.4% | 73.8%  21.4%  2.4%  0  0  2.4% | 0  45%  42.5%  7.5%  2.5%  2.5% |
| **Gender (in percent)**  Female  Male  Other | 73.6%  24.5%  2% | 58.2%  41.8%  0 | 83.3%  11.9%  4.8% | 82.5%  15%  2.5% |
| **Experience with personal mental illness**  Yes  No  I don’t know | 50.9%  49.1%  0 | 29.1%  70.9%  0 | 35.7%  59.5%  4.8% | 37.5%  62.5%  0 |
| **Experience with mental illness of others**  Yes  No  I don’t know | 96.2%  1.9%  1.9% | 87.3%  5.5%  7.2% | 95.2%  4.8%  0 | 87.5%  7.5%  5% |
| **Outcomes*** |  | | | |
| Implicit mental illness stigma: SC-IAT D-Score | -0.02 *(0.06)* | -0.21 *(0.35)* | 0.02 *(0.47)* | -0.12 *(0.42)* |
| Explicit public mental illness stigma: VASI | 5.62 *(1.22)* | 6.35 *(1.22)* | 5.71 *(1.13)* | 5.35 *(1.14)* |
| Explicit self-stigma of seeking help: SSOSH | 2.16 *(0.60)* | 2.15 *(0.60)* | 2.12 *(0.57)* | 1.93 *(0.63)* |
| Desire for social distance towards PLMI: SDS | 4.32 *(0.50)* | 3.90 *(0.72)* | 4.23 *(0.60)* | 4.21 *(0.54)* |
| Social desirability: KSE-G  *Maximization of positive qualities: PQ+*  *Minimization of negative qualities: NQ-* | 3.21 *(0.75)*  2.38 *(0.86)* | 3.12 *(0.80)*  2.12 *(0.94)* | 3.33 *(0.65)*  2.24 *(0.81)* | 3.22 *(0.75)*  2.65 *(1.08)* |

**Supplementary Table S1: Participant characteristics, means and standard deviations of the outcomes**

*Notes.* *Mean (Standard Deviation); D-Score as outcome variable of Single Category Implicit Association Task (SC-IAT); VASI = Value-Based Stigma Inventory; SSOSH = Self-Stigma of Seeking Help questionnaire; SDS = Social Distance Scale; KSE-G = Social Desirability Scale “Kurzskala Soziale Erwünschtheit-Gamma”.
